# Perceived parenting and psychological well-being in UK ethnic minority adolescents

**DOI:** 10.1111/j.1365-2214.2010.01115.x

**Published:** 2010-09

**Authors:** M J Maynard, S Harding

**Affiliations:** Medical Research Council/Chief Scientists Office Social and Public Health Sciences UnitGlasgow, UK

**Keywords:** adolescence, ethnic groups, mental health, parenting

## Abstract

**Background:**

Warm, caring parenting with appropriate supervision and control is considered to contribute to the best mental health outcomes for young people. The extent to which this view on ‘optimal’ parenting and health applies across ethnicities, warrants further attention. We examined associations between perceived parental care and parental control and psychological well-being among ethnically diverse UK adolescents.

**Methods:**

In 2003 a sample of 4349 pupils aged 11–13 years completed eight self-reported parenting items. These items were used to derive the parental care and control scores. Higher score represents greater care and control, respectively. Psychological well-being was based on total psychological difficulties score from Goodman's Strengths and Difficulties Questionnaire, increasing score corresponding to increasing difficulties.

**Results:**

All minority pupils had lower mean care and higher mean control scores compared with Whites. In models stratified by ethnicity, increasing parental care was associated with lower psychological difficulties score (better mental health) and increasing parental control with higher psychological difficulties score within each ethnic group, compared with reference categories. The difference in psychological difficulties between the highest and lowest tertiles of parental care, adjusted for age, sex, family type and socio-economic circumstances, was: White UK =−2.92 (95% confidence interval −3.72, −2.12); Black Caribbean =−2.08 (−2.94, −1.22); Nigerian/Ghanaian =−2.60 (−3.58, −1.62); Other African =−3.12 (−4.24, −2.01); Indian =−2.77 (−4.09, −1.45); Pakistani/ Bangladeshi =−3.15 (−4.27, −2.03). Between ethnic groups (i.e. in models including ethnicity), relatively better mental health of minority groups compared with Whites was apparent even in categories of low care and low autonomy. Adjusting for parenting scores, however, did not fully account for the protective effect of minority ethnicity.

**Conclusions:**

Perceived quality of parenting is a correlate of psychological difficulties score for all ethnic groups despite differences in reporting. It is therefore likely that programmes supporting parenting will be effective regardless of ethnicity.

## Introduction

Social context such as material disadvantage and discrimination, and conversely social support, as explanations of ethnic differences in adult mental health have been examined in the UK (Sproston & Nazroo 2002) and elsewhere (Harris *et al*. 2006; Veling *et al*. 2007; Williams *et al*. 2007). Less is known about ethnic differences in adolescent mental health. Extant UK literature indicates that better mental health scores reported for Black African (Maynard *et al*. 2007), Indian (Meltzer *et al*. 2000; Green *et al*. 2005) and Bangladeshi (Stansfeld *et al*. 2004) origin adolescents compared with Whites are not explained by socio-economic circumstances, family type or social support (Klineberg *et al*. 2006; Maynard *et al*. 2007). It is possible, however, that the quality of family interactions may be more important, buffering the adverse affects of social deprivation on health (Farrell *et al*. 1995; Glendinning *et al*. 2000; Maynard & Harding 2010). The effect on adolescent and later well-being of adverse emotional events in early life such as death or separation of parents is well known (Offord & Bennett 2002) but there has been less focus on the chronic emotional stress of poor parent–child relationships (Aquilino & Supple 2001; Patton *et al*. 2001; Stewart-Brown *et al*. 2005). The extent to which the long-held view on ‘optimal’ authoritative parenting – high levels of support, adequate monitoring balanced with opportunities to develop autonomy and avoidance of harsh punishment (Baumrind 1968) – benefits young people across ethnic groups has been explored in the USA (Aquilino & Supple 2001; Walker-Barnes & Mason 2001; Amato & Fowler 2002; Lansford *et al*. 2004; Vendlinski *et al*. 2006) and warrants further interrogation in the UK (Phoenix & Husain 2007). In US studies lower parent–child openness was associated with greater child anxiety and depression symptoms (Vendlinski *et al*. 2006) and physical discipline and ‘no-nonsense’ parenting with more conduct problems (Walker-Barnes & Mason 2001; Lansford *et al*. 2004) among White compared with Black Americans, the putative mechanism being differences in normativeness of these family attributes (Vendlinski *et al*. 2006). We use measures of young peoples' subjective reports of their parenting and psychological well-being to examine the hypothesis that authoritarian parenting is more common for ethnic minority than White UK adolescents but is not detrimental to mental well-being among minorities.

## Methods

### The Determinants of Adolescent Social wellbeing and Health study

The sample was recruited from 51 schools in 10 London boroughs. Pupils in Years 7 and 8 (aged 11–13 in 1st and 2nd years of secondary school), in randomly selected mixed ability classes, were invited to join the study. Full details of the sampling strategy have previously been reported (Harding *et al*. 2007). Approval was obtained from the Multi-centre Research Ethics Committee and Local Education Authorities. Parents were provided with information packs prior to the start of the study, via head teachers and a parental opt-out consent procedure employed. Active consent was required from pupils. The pupil response rate was 83%. Further details of the study can be found at http://dash.sphsu.mrc.ac.uk/

### Perceived parenting style

Each pupil self-completed a questionnaire on their health and social circumstances. Questionnaires were completed in the classroom under exam conditions with researchers available to assist students with comprehension of the questions. Parenting was measured using the eight-item Parental Bonding Instrument (Klimidis *et al*. 1992). Each item is scored on a 4-point scale from which two variables are derived: ‘care’ (from the items ‘help me as much as I need’, ‘are loving’, ‘understand my problems and worries’ and ‘make me feel better when I am upset’) and ‘control’ (‘let me do the things I like doing’, ‘like me to make my own decisions’, ‘try to control everything I do’ and ‘treat me like a baby’). Higher scores represent the perception of greater care and greater control, respectively.

### Ethnicity and potential confounders

The questionnaire also covered information on ethnicity, household composition and standard of living items. Age was determined from reported date of birth. Ethnicity was identified by combining self-reported ethnicity, having at least one parent with the same ethnicity and having at least three grandparents who were born in home countries. Access to 17 standard of living items (in tertiles) was used as a proxy measure of socio-economic status (SES). Multidimensional measures appear to capture disadvantage in ethnic minorities more so than traditional measures, such as class, because of disruption of life chances or the inability to make use of qualifications gained in home countries (Nazroo 1997). Our proxy measure of SES correlates well with parental employment status (Harding *et al*. 2008) within every minority group. It also reflects similar ethnic patterns (e.g. Pakistanis and Bangladeshis more socio-economically disadvantaged than Indians) of individual level measures in other sources (e.g. social class in the 2001 census http://www.ons.gov.uk/census/index.html). Family type is defined as two-parent (living with both biological parents), reconstructed (living with one biological parent and one other in the parental role, e.g. step-parent), lone-parent (living with one parent only) and ‘other’ (no parent in the family home, e.g. living with other relatives, foster carers, etc.).

### Outcome measurement

Psychological well-being was measured with the 25-item Strengths and Difficulties Questionnaire (SDQ), a validated behavioural screening tool providing coverage of children's behaviour, emotions and peer relations (Goodman 1997). The SDQ was completed by the pupils as part of the health and social circumstances questionnaire, under the conditions detailed above. It comprises five scales of five items each rated on a 3-point scale. The scales are emotional symptoms, conduct problems, hyperactivity, peer problems and pro-social behaviour. A total psychological difficulties score ranging from 0 to 40, representing increasing difficulties, is derived by summing scores on the first four of these subscales (http://www.sdqinfo.com).

### Statistical methods

The analysis is based on some of the main ethnic minority groups in the UK: 929 Black Caribbean, 612 Nigerian and Ghanaian, 468 Other African (mostly Somalis and Eritreans), 492 Indian, 402 Pakistani, 219 Bangladeshi and 1227 White UK boys and girls who completed the SDQ. The Bangladeshi group was too small to examine separately and was combined with the Pakistanis. Both of these groups are distinctly different from Indians being more deprived (as described above) and with a worse health profile in adulthood (Nazroo 1997; Harding & Balarajan 2001). The remaining DASH (Determinants of Adolescent Social wellbeing and Health) participants of other ethnicities [e.g. Mixed, White Other (mainly Eastern Europeans and Irish) and Other (including Eastern Asians)] are not included in these analyses as they are not of sufficient sample size. Tertiles of care and of control (based on the distributions in the whole sample) were used to ensure that the extremes of the distribution of both scales could be identified. Linear regression was used to explore the association between tertiles of the parenting scores and mean psychological difficulties score. Regression models were stratified by ethnicity to examine the effect of the care and control scores on mean total difficulties within each group. We then went on to formally compare ethnic groups (by including ethnicity in the models) with parenting scores, SES and family type added stepwise to these models to assess their moderating effects on psychological difficulties score. First and second order interaction tests were carried out to examine possible interactions between parenting, ethnicity, gender, family type and SES in their association with psychological well-being.

## Results

Table 1 shows the characteristics of the sample by ethnic group. Black Caribbeans were less likely and South Asians more likely than Whites to be in two-parent households. Minority groups, with the exception of the Indians, were more disadvantaged than their White peers. Within ethnic groups, the distribution of the social variables (family type and SES) was largely similar across the distribution of both parenting scores. Exceptions were among the Whites and the Black Caribbeans. There was a greater proportion of disadvantaged Black Caribbeans in the lowest tertile of care compared with the highest tertile (52% vs. 40%), and a greater proportion of better-off Black Caribbeans in the lowest tertile of control compared to the highest (32% vs. 21%). Whites in the highest tertile of control were less likely to come from two parent families (50% vs. 64%) and more likely to be disadvantaged (47% vs. 34%) compared to those in the lowest tertile of control (not shown in Table). All minority groups reported lower mean care score and higher control score than Whites.

**Table 1:** Characteristics of the sample and distribution of the parental ‘care’ and ‘control’ scores

	**White UK**	**Black Caribbean**	**Nigerian/Ghanaian**	**Other African**	**Indian**	**Pakistani/Bangladeshi**
*n*	1227	929	612	468	492	621
% in lone-parent households	23	39	26	31	5	10
% least advantaged (3rd tertile of socio-economic status score)	36	41	49	46	30	44
Age- and sex-adjusted mean total difficulties score (95% CI)	11.55 (11.26, 11.84)	11.30 (10.96, 11.63)	10.48 (10.07, 10.89)*	10.57 (10.10, 11.04)*	9.89 (9.43, 10.35)*	10.71 (10.30, 11.12)*
Age- and sex-adjusted mean (SD) parental care	14.31 (0.06)	13.97 (0.09)*	13.72 (0.11)*	13.82 (0.14)*	13.95 (0.12)*	14.0 (0.11)*
Age- and sex-adjusted mean (SD) parental control	7.28 (0.06)	7.83 (0.08)†	8.35 (0.10)†	8.19 (0.12)†	8.09 (0.10)†	8.20 (0.09)†

* Significantly lower than White UK.

† Significantly higher than White UK (*P* < 0.05).

### The association between parenting scores and psychological difficulties, within each ethnic group

Table 2 shows the effect of increasing tertiles of parental care and parental control on psychological difficulties score in models stratified by ethnic group. There were no significant gender interactions in the associations; therefore, all analyses include both boys and girls, adjusting for sex.

**Table 2:** Regression coefficients (95% CI) for the effect of increasing tertiles of parental care and of parental control on psychological well-being (total difficulties score – TDS) within each ethnic group, adjusted for age, sex, family type and socio-economic status

	**White UK**	**Black Caribbean**	**Nigerian/Ghanaian**	**Other African**	**Indian**	**Pakistani/Bangladeshi**
Care						
Tertile 1 (reference): sex- and age-adjusted mean TDS (95% CI)	13.64 (12.79, 14.48)	12.81 (12.02, 13.60)	12.29 (11.43, 13.15)	12.77 (11.97, 13.58)	11.26 (10.22, 12.30)*	13.09 (11.85, 14.32)
Model 1						
Tertile 2	−1.66 (−2.60, −0.73)†	−1.51 (−2.54, −0.48)†	−2.18 (−3.34, −1.02)†	−2.13 (−3.50, −0.76)†	−0.53 (−2.08, 1.09)	−1.78 (−3.08, −0.48)†
Tertile 3	−2.97 (−3.78, −2.17)†	−2.08 (−2.93, −1.24)†	−2.74 (−3.74, −1.74)†	−3.03 (−4.12, −1.94)†	−2.49 (−3.80, −1.19)†	−3.05 (−4.16, −1.94)†
Model 2						
Tertile 2	−1.68 (−2.61, −0.75)†	−1.47 (−2.51, −0.43)†	−2.18 (−3.33, −1.02)†	−2.19 (−3.57, −0.82)†	−0.77 (−2.33, 0.80)	−1.91 (−3.19, −0.63)†
Tertile 3	−2.92 (−3.72, −2.12)†	−2.08 (−2.94, −1.22)†	−2.60 (−3.58, −1.62)†	−3.12 (−4.24, −2.01)†	−2.77 (−4.09, −1.45)†	−3.15 (−4.27, −2.03)†
Control						
Tertile 1 (reference): sex- and age-adjusted mean TDS (95% CI)	10.64 (10.15, 11.13)	10.07 (9.52, 10.61)	8.67 (8.07, 9.28)*	9.02 (8.28, 9.75)*	8.65 (7.96, 9.34)*	9.92 (9.25, 10.58)
Model 1						
Tertile 2	1.47 (0.82, 2.12)‡	1.43 (0.66, 2.19)‡	1.23 (0.29, 2.18)‡	1.93 (0.86, 3.00)‡	1.44 (0.35, 2.53)‡	0.22 (−0.73, 1.16)
Tertile 3	4.29 (3.39, 5.19)‡	3.75 (2.87, 4.63)‡	4.15 (3.16, 5.13)‡	3.35 (2.21, 4.48)‡	2.87 (1.71, 4.03)‡	2.31 (1.29, 3.33)‡
Model 2						
Tertile 2	1.45 (0.80, 2.10)‡	1.41 (0.64, 2.18)‡	1.38 (0.46, 2.31)‡	1.98 (0.91, 3.05)‡	1.44 (0.34, 2.54)‡	0.25 (−0.69, 1.20)
Tertile 3	4.14 (3.24, 5.04)‡	3.71 (2.82, 4.60)‡	4.15 (3.19, 5.11)‡	3.36 (2.21, 4.51)‡	2.93 (1.77, 4.09)‡	2.35 (1.34, 3.37)‡

Adjusted for: Model 1 = age and sex, Model 2 = age, sex, socio-economic status and family type.

* Significantly lower than White UK mean.

† Significantly lower than reference mean within the same ethnic group.

‡ Significantly higher than reference mean within the same ethnic group (*P* < 0.05).

### Care

With the exception of the Indians for whom mean psychological difficulties score in the reference category (tertile 1, least care) was significantly lower than Whites, reference mean psychological difficulties score was similar across groups. There was a significant linear decrease in difficulties score (better mental health) with increasing care for all ethnic groups with the exception of the Indians where the association was seen in the top tertile only. The pattern of association was similar before and after adjustment for family type and SES.

### Control

In the reference category of control (tertile 1, least control) mean psychological difficulties score was significantly lower than Whites for Nigerian/Ghanaians, Other Africans and Indians. There was a pattern of increasing psychological difficulties score (poorer mental health) with increasing control for all groups. The association was significant in the top tertile only for Pakistani/Bangladeshis and linear for all other groups. As with the care score the pattern of association was not materially altered by full adjustment for social factors.

### Ethnicity and psychological difficulties, stratified by parenting scores

Formally comparing ethnic groups (i.e. with ethnicity included in regression models), minority groups had significantly lower psychological difficulties score than Whites (Table 3). In these models there was also a suggestion of an interaction between ethnicity and both parenting scores although no *P*-values were <0.05. To explore this further, models were stratified by tertiles of the two parenting scores. The regression coefficients were adjusted for sex, family type, SES and ethnicity.

**Table 3:** Regression coefficients* (95% CI) for differences in mean total difficulties scores (TDS) for minority groups compared with White UK (reference group) within tertiles of parental care and tertiles of parental control

	**White UK (reference mean TDS)**	**Black Caribbean**	**Nigerian/Ghanaian**	**Other African**	**Indian**	**Pakistani/Bangladeshi**
Overall	11.58 (11.24, 11.92)	−0.53 (−1.00, −0.06)†	−1.05 (−1.58, −0.51)†	−1.22 (−1.79, −0.64)†	−1.30 (−1.88, −0.71)†	−0.75 (−1.29, −0.20)†
Care						
Tertile 1 (low)	13.71 (12.91, 14.53)	−1.12 (−2.21, −0.04)†	−1.36 (−2.53, −0.18)†	−1.23 (−2.51, 0.05)	−1.52 (−2.84, −0.20)†	−0.98 (−2.21, 0.24)
Tertile 2	11.92 (11.27, 12.56)	−0.95 (−1.93, 0.02)	−1.85 (−2.90, −0.80)†	−1.46 (−2.70, −0.21)†	−0.59 (−1.83, 0.64)	−0.66 (−1.75, 0.43)
Tertile 3 (high)	10.73 (10.34, 11.12)	−0.30 (−0.90, 0.31)	−1.08 (−1.79, −0.37)†	−1.12 (−1.88, −0.36)†	−1.51 (−2.24, −0.78)†	−0.82 (−1.50, −0.13)†
Control						
Tertile 1 (low)	10.54 (10.14, 10.95)	−0.69 (−1.31, −0.07)†	−1.77 (−2.52, −1.01)†	−1.62 (−2.46, −0.79)†	−1.70 (−2.49, −0.90)†	−0.68 (−1.44, 0.09)
Tertile 2	11.97 (11.46, 12.48)	−0.58 (−1.36, 0.20)	−1.95 (−2.81, −1.09)†	−1.04 (−1.99, −0.08)†	−1.63 (−2.57, −0.69)†	−1.70 (−2.54, −0.85)†
Tertile 3 (high)	14.86 (13.92, 15.80)	−1.35 (−2.62, −0.08)†	−1.81 (−3.10, −0.51)†	−2.59 (−3.99, −1.19)†	−2.79 (−4.23, −1.35)†	−2.15 (−3.47, −0.84)†

* Adjusted for age, sex, ethnicity, socio-economic status and family type.

† Significantly lower than White UK (*P* < 0.05).

### Care

Within the highest tertile of care, minority ethnicity was associated with a protective effect on mental health for most groups compared with Whites. However, within the lowest category of care (tertile 1) psychological difficulties score was also significantly lower for Black Caribbeans, Nigerian/Ghanaians and Indians (Table 3).

### Control

In tertile 1 of control all minority groups except Pakistani/Bangladeshis had significantly lower psychological difficulties score than Whites. This protective effect of minority ethnicity remained in tertile 2 of control for Nigerian/Ghanaians, other Africans and Indians. Consistently across minority groups, in the categories of least autonomy (tertile 3) psychological difficulties score was lower than for Whites (Table 3).

To further unpick this finding the association between ethnicity and tertile 3 of the control score was examined within family type and SES strata (Table 4). All minority groups in tertile 3 of parental control and from two-parent families had better mental health scores than Whites. There was also an independent protective effect for Other Africans in lone-parent families and for all groups (except Black Caribbeans) in the less advantaged tertiles of the SES score.

**Table 4:** Regression coefficients (95% CI) for differences in mean total difficulties scores (TDS) for minority groups compared with White UK (reference group) within tertile 3 of the control score by family type* and socio-economic status†

	**White UK (reference mean TDS)**	**Black Caribbean**	**Nigerian/Ghanaian**	**Other African**	**Indian**	**Pakistani/Bangladeshi**
Family type						
Two-parent	14.60 (13.28, 15.92)	−2.22 (−4.39, −0.04)‡	−2.45 (−4.22, −0.67)‡	−2.20 (−4.26, −0.15)‡	−3.25 (−4.98, −1.52)‡	−2.36 (−3.98, −0.73)‡
Lone-parent	15.76 (14.10, 17.42)	0.95 (−2.30, 1.09)	−2.09 (−4.38, 0.20)	−2.61 (−5.06, −0.17)‡	−0.79 (−5.66, 4.07)	−2.54 (−5.56, 0.47)
Socio-economic status score						
Most advantaged tertile	14.30 (12.67, 15.94)	−1.03 (−3.44, 1.37)	−0.82 (−3.44, 1.80)	−0.84 (−3.86, 2.18)	−1.82 (−4.31, 0.67)	−0.66 (−2.99, 1.67)
Middle tertile	17.50 (15.28, 19.71)	−5.14 (−8.44, −1.84)‡	−4.12 (−7.12, −1.13)‡	−4.95 (−8.43, −1.47)‡	−6.96 (−10.15, −3.76)‡	−4.59 (−7.74, −1.43)‡
Least advantaged tertile	14.74 (13.45, 16.04)	−1.30 (−3.04, 0.43)	−2.42 (−4.16, −0.67)‡	−2.44 (−4.35, −0.54)‡	−2.46 (−4.65, −0.26)‡	−2.40 (−4.31, −0.49)‡

* Adjusting for age, sex and socio-economic status.

† Adjusting for age, sex and family type.

‡ Significantly lower than White UK (*P* < 0.05).

### Ethnicity and psychological difficulties, adjusting for parenting scores

**Figure 1:**
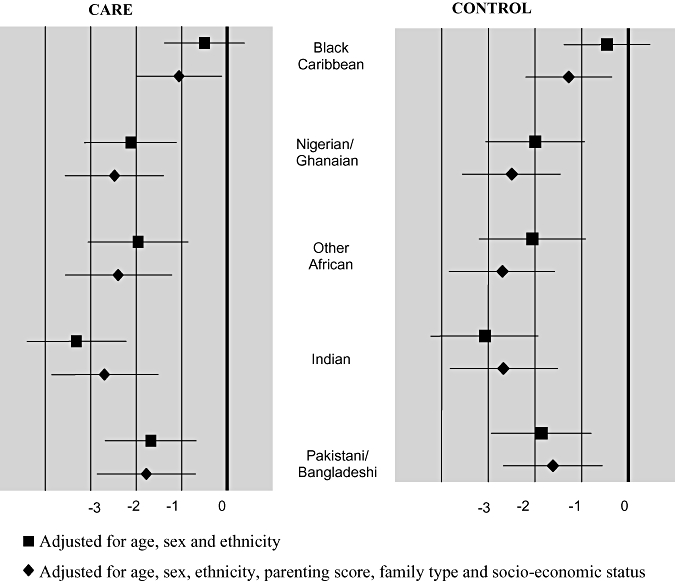
Ethnic differences in mean total difficulties scores (minority groups compared with White UK). Regression coefficients and 95% CI (reference: mean total difficulties score for White UK).

The effects of adjustment for the parenting scores in models including ethnicity are shown in Fig. 1. In most cases this adjustment resulted in only small shifts in the association between ethnicity and psychological difficulties score, significantly increasing the difference in psychological difficulties score among Black Caribbeans relative to Whites. Adjusting for the care score slightly attenuated the association for the South Asian groups. Care and control remained significant independent correlates for psychological difficulties in these models.

## Discussion

This study is the first in the UK to examine the effect of perceived parenting style on adolescent psychological well-being in an ethnically diverse sample. We report an association between increasing care score and better mental health scores consistently within all groups. The highest tertile of control was associated with poor mental health for all groups, contrary to our hypothesis. Reverse causality cannot be ruled out but elsewhere parental style has predicted adolescent behavioural outcomes in longitudinal analysis (Amato & Fowler 2002). We will explore this issue in our sample with analysis of follow-up data currently in preparation. Between ethnic groups, the pattern of relatively better mental health for minority groups compared with Whites was apparent even in categories of low care for some groups and low autonomy for all minority groups. Adjusting for parenting scores, however, did not fully account for the better mental health scores among minorities.

A potential drawback of the study is that of shared method variance. Self-reports of abuse and neglect are thought to be valid and accurate even in retrospect (Giovannoni 1989) and social deprivation and stressful events in early life are known to effect mental health in adolescence (Glendinning *et al*. 2000) and in later life (Stewart-Brown *et al*. 2005). Less is known about the validity of self-reports of everyday interactions between children and adults (Stewart-Brown *et al*. 2005). Others have suggested, however, that social desirability bias is less likely in child vs. parental reports of parenting approach (Aquilino & Supple 2001). A further complication is that adolescence is a time when relationships between parents and children become increasingly volatile and recent unusual conflict or harmony may have had an influence on reporting at the individual point in time of data collection. We are also unable to determine the relative influence of mothers' and fathers' parenting or to resolve the potential reporting difficulties of those with two parents with very different parenting styles.

High profile cases of abuse and overrepresentation of some minority groups receiving formal child protection services (Barn 2001; Department for Education and Skills 2005) reinforce the idea that minority families use particularly harsh parenting (Barn *et al*. 2006). Studies of parenting among families from some of the main minority ethnic communities in the UK report emphasis on a culture of respect and preference for a collective responsibility model among some groups, but also a range of parenting practices (Dosanjh & Ghuman 1996; Barn *et al*. 2006). In our study the excess reporting by minorities compared with Whites of what is usually considered less positive parenting (relatively lower care and lower autonomy) does suggest differences in parenting between ethnic groups. It is possible, however, that there are cultural or other norms influencing interpretation and/or significance of the parenting items. For example, if greater parental control is considered normative in more traditional families it may be less negatively associated with mental health outcomes for the children compared with those from less traditional homes. Furthermore, others contest the notion that authoritative parenting style is optimal if it needs to be ‘supplemented by alternative explanations for some groups and not others’ (Phoenix & Husain 2007, p. 13). In our study, that the pattern of relatively better mental health, even with stricter parenting, was seen among the less advantaged as well as two-parent families warrants further attention, but supports a cultural explanation. Multidimensional measures such as our composite SES score may be more appropriate for assessing disadvantage in ethnic minorities (as noted above). With the absence of other aspects of SES such as education in the social class adjustment, nonetheless, it is possible that social class confounding may remain. There could also be ethnic differences in the availability of wider social support that may buffer the effect of parent–child relationships.

It appears that the mechanism by which perceived parenting is associated with psychological difficulties score is the same across ethnic groups in our study. There is some evidence that programmes supporting helpful parenting can improve child well-being and behaviour as well as parental mental health across ethnic groups (Barlow *et al*. 2004). Our analyses concur that such programmes are likely to be effective regardless of ethnicity.

**Key messages**

- Studies examining the role of parenting in adolescent mental health in the UK have not previously been carried out in large, ethnically diverse samples.
- Despite heterogeneity in reporting of perceived parenting, low care and high control scores were associated with poorer mental health within each ethnic group in this sample of 11- to 13-year-olds. It is likely that parenting programmes would be effective regardless of ethnicity.
- Between ethnic groups, minorities had relatively better mental health compared with Whites, even in environments of low care and autonomy. Adjusting for parenting scores, however, did not explain the protective effect of minority ethnicity.
